# Multi-User Precoder Designs for RGB Visible Light Communication Systems

**DOI:** 10.3390/s20236836

**Published:** 2020-11-29

**Authors:** Roser Viñals, Olga Muñoz, Adrián Agustín, Josep Vidal

**Affiliations:** 1 The Department of Signal Theory and Comunications, Universitat Politècnica de Catalunya (UPC); 08034 Barcelona, Spain; roser.vinalsterres@epfl.ch (R.V.); adrian_agustin@telefonica.net (A.A.); josep.vidal@upc.edu (J.V.); 2 Signal Processing Laboratory (LTS5), École Polytechnique Fédérale de Lausanne (EPFL); 1015 Lausanne, Switzerland

**Keywords:** visible light communications, multi-user, linear precoding, adaptive modulation, zero forcing, weighted sum-rate maximization, Red-Green-Blue, RGB-LEDs

## Abstract

In this paper, we design linear precoders for the downlink of a visible light communication (VLC) system that simultaneously serves multiple users. Instead of using phosphor-coated white light-emitting diodes (PWLEDs), we focus on Red-Green-Blue light-emitting diodes (RGB-LEDs) that allow modulating three separate data streams on the three primary colors of the RGB-LEDs. For this system, we design a zero-forcing (ZF) precoder that maximizes the weighted sum rate for a multilevel pulse amplitude modulation (M-PAM). The precoding design in RGB-based systems presents some challenges due to the system constraints, such as the limited power, the non-negative amplitude constraints per light-emitting diode (LED), and the need to guarantee white light emission while transmitting with RGB-LEDs. For comparison purposes, we also consider the ZF design for a PWLED-based system and evaluate the performance of both a PWLED- and an RGB-based system.

## 1. Introduction

Due to the limitation in the availability of spectrum and the millimeter wave (mmWave) signals propagation problems, short-range Visible Light Communications (VLC) has received much attention from the research community, experiencing significant advances in the last years. VLC technology can operate in the non-regulated visible light spectrum of approximately 400 THz using light-emitting diodes (LEDs) and photodiodes (PD) as transmitters and receivers, respectively, for downlink (DL) transmissions. LEDs can switch at different light intensity levels at a high-speed rate but imperceptible to the human eye. Because of that, off-the-shelf LEDs commonly used for illumination can also act as communication transmitters, being this one of the most exciting features of VLC systems [1].

**Figure 1:**
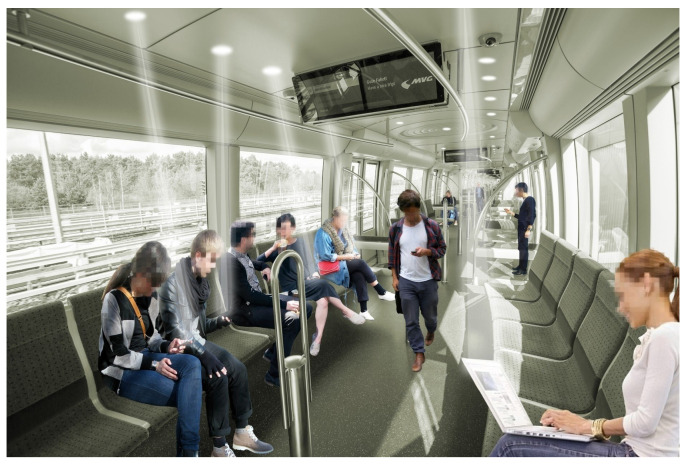
An illustrative scenario for a Visible Light Communications (VLC)-based multiuser downlink data transmission.

Figure 1 shows an illustrative scenario of application. In the figure, several passengers in a rail wagon receive the information transmitted by a set of LEDs placed in the wagon ceiling. An indoor broadcast system such as this requires the design of VLC techniques dealing with the multi-user (MU) interference while meeting with the specificities of the VLC signals. Combating the DL interference has been an active area of research of Radio Frequency (RF) systems for many years. However, applying the available RF techniques directly to VLC systems is not possible because of the differences between RF and VLC systems that critically affect the system design (see Table 1). First, in VLC systems, the data are encoded in the signal amplitude using an intensity-modulated/direct-detection (IM/DD) modulation. In contrast, modulations in RF systems use the phase, amplitude, and signal frequency. Second, as the waveform transmitted is conveyed by the optical power, the signal must be real and non-negative. Consequently, the VLC average transmitted power depends on the time-average of the channel input, while the RF average transmitted power depends on the time-average of the square of the channel input.

In the last years, the research community has made many efforts to deal with the above issues while taking advantage of the RF MU systems’ vast knowledge. In [3,4,5,6,7,8,9,10,11], the authors design MU techniques for VLC systems with white phosphor LEDs. To produce white light, the phosphor-coated LED (PWLED) combines a blue LED with a yellow phosphor coating. Although the PWLED is low cost and commercially available, the phosphor coating limits the LED’s velocity to switch between different light intensity levels. This limitation reduces the transmission bandwidth (BW) to a few MHz [12,13]. In contrast, a Red-Green-Blue (RGB) LED provides higher bandwidths and can modulate the three colors individually (the receiver can separate them through optical filters). Furthermore, as there is no limitation due to the phosphor coating [14], each color channel of an RGB-LED provides a typical BW between 10 and 20 MHz [15,16].

Although RGB-LEDs can, in principle, provide improved performance, it is not obvious how to design transmitter precoders in multi RGB-based systems or to predict the performance achieved. RGB-based systems modify the intensity of the different color LEDs according to the information-bearing signal, for instance, using color Shift Keying (CSK) modulation [17] as in [16,18,19,20,21,22,23,24]. Other RGB system designs allow the RGB system design to adapt to any target color [25,26,27,28,29,30]. Among previous works, the works in [23,24,26,27] deal with MU scenarios. While in [23,26,27], the authors add MU capabilities through a time-based multiplexing approach, in [24], the authors propose a MU joint constellation design for VLC downlink broadcast channels. MU precoding has been shown to be a much more efficient approach than TDMA [9] for PWLED-based VLC systems and allows at the same time to keep an independent polar multi-level pulse amplitude modulation (M-PAM) for each user, which may simplify the detection process. In [25], an iterative Mean Square Error (MSE) minimization algorithm is proposed for designing a MIMO precoder and equalizer for single-user scenarios using multi-color LEDs. However, the approach in [25] does not ensure the maximization of the rate or the minimization of the bit error rate, particularly in MU scenarios.

Unlike previous approaches, we focus on MU scenarios and precoding schemes for RGB-LEDs that can cancel MU interference and adapt the users’ rate to channel conditions. We design such strategies by formulating non-trivial optimization problems that we solve through convex optimization techniques. To that end, we follow similar procedures as those followed in [9], a preliminary work of ours for the more simple case of white phosphor LEDs (so users receive only one data stream). In contrast to the work in [9], in this paper we focus on RGB-based systems. Working with RGB-based systems requires a new formulation of the precoding design problem, which is more general and challenging.

### 1.1. Organization

We introduce the system model in Section 2. Then, in Section 3, we formulate the optical and electrical constraints for the downlink of an RGB based VLC system. For the described system, and considering the RGB based system’s constraints, we design in Section 4 multi-user precoders that take a zero-forcing (ZF) approach to cancel multi-user interference. Besides, we show in Section 5 that the RGB precoder design can be particularized to the simpler white phosphor LED case, leading to the solution we presented in [9]. Finally, in Section 6, we evaluate the proposed approaches and provide conclusions in Section 7.

### 1.2. Notation

We denote vectors and matrices with bold lower and upper case letters, respectively. For matrices, we indicate the transpose, inverse, and pseudo-inverse with the superscript ${\left(\cdot \right)}^{T}$, ${\left(\cdot \right)}^{-1}$, and ${\left(\cdot \right)}^{†}$, respectively. We use I to indicate an identity matrix and $1_{N}$ to indicate an all 1s column vector of *N* elements. Throughout the paper, we also use E[·] and ${\left||\cdot |\right|}_{p}$ to indicate expected value and the *p*-norm. Finally, we use the symbol ⊗ for the Kronecker product.

## 2. System Model

In this section, we present the downlink multi-user RGB VLC system considered in this paper. In this system, the transmitter is composed of *N* RGB units of three color LEDs able to switch light intensity fast enough without the limitation of the phosphor-coated LEDs (i.e., each RGB unit is composed of a red, a green, and a blue LED co-located together). The L=3N LEDs jointly transmit D=3K streams to *K* users. Therefore, each user receives three streams, one for each color channel. To separate the three color bands, each user is equipped with one receiver consisting of three photodiodes (PDs) with a color filter (i.e., a narrowband optical filter centered at the desired wavelength of the corresponding red, green, or blue band).

Let $x\in R^{L\times 1}$ be the optical signal vector transmitted by the whole set of L=3N LEDs, $x={\left[x_{R}^{T},x_{G}^{T},x_{B}^{T}\right]}^{T}$. An individual LED, l=1,…,L, can be identified by the index of the set the LED belongs to and the specific color of the LED, i.e., l=(c−1)·N+n with c=1,2,3 if the color is R, G, B, respectively; and n=1,…,N. We can express the transmitted signal as

**Figure 2:**
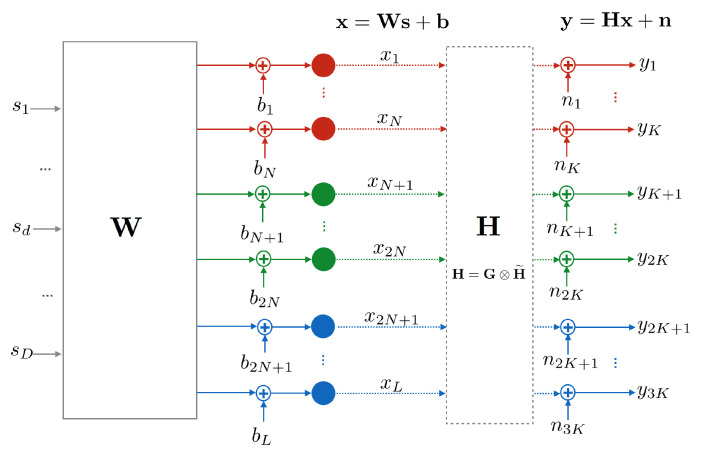
Block diagram of the system considered. A total of L=3N LEDs transmit D=3K symbol streams over *L* LEDs to *K* users with RGB-photodiodes.

(1) x=Ws+b,

where **$W=\left[w_{1},w_{2},\ldots ,w_{D}\right]\in R^{L\times D}$** is the precoding matrix, $s\in R^{D\times 1}$ is the vector of symbols transmitted at a single time slot (time dependency is omitted for simplicity in the formulation), and $b={\left[b_{R}^{T},b_{G}^{T},b_{B}^{T}\right]}^{T}\in R^{L\times 1}$ is the bias vector that accounts for the constant DC current applied to each LED. The block diagram for the system model is shown in Figure 2.

We will consider that the symbols belong to an *M*-PAM modulation normalized to the range [−1,1], where *M* indicates the size of the alphabet. Compared to other single carrier modulations such as Pulse Position Modulation (PPM) or binary on–off keying (OOK), M-PAM presents better spectral efficiency [1], and therefore it is relevant in our study. The electrical power for this modulation, obtained through the second-order moment of the symbols [1], is

(2) $$P_{s,\;elec}=E\left\{s^{2}\right\}=\frac{1}{3}\frac{\left(M+1\right)}{\left(M-1\right)}.$$

Note that the LEDs’ nonlinearities that may affect M-PAM can be combated by conditioning the transmitted signals within the transmitter’s linear dynamic range with the control of the signal variance and the constant DC current, as we will see in Section 3.

**Figure 3:**
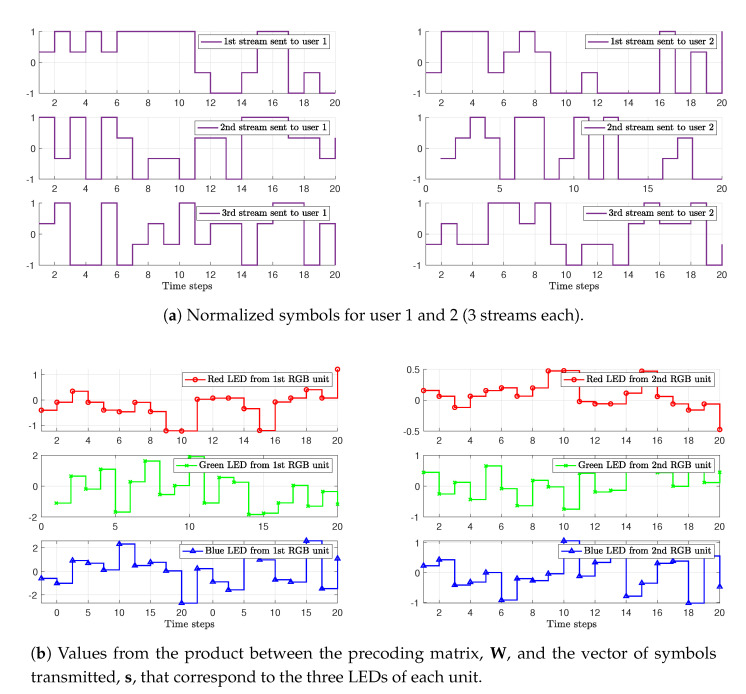

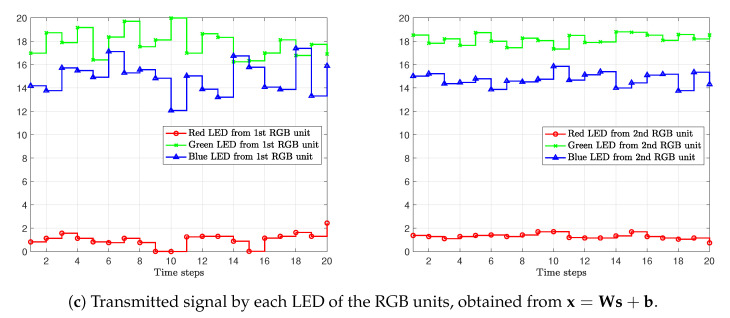
Illustration of the signals generated in a system with N=2 RGB units, each one with one red, one green, and one blue LED, and K=2 users, both supporting 4-PAM: (**a**) users’ streams (3 symbols stream per user), (**b**) linear combination of the users’ streams to be sent by each LED, and (**c**) final optical power emitted by each LED. The R, G, and B primaries correspond to set 2 specified in Table 3.

Consider as an example a system with N=2 RGB units, each one with one red LED, one green LED, and one blue LED, and K=2 users, both supporting 4-PAM. Each of the six LEDs will transmit a linear combination of D=3K=6 streams, i.e., three streams per user. Figure 3a,b show the 4-PAM users’ streams and the linear combination of the six streams to be sent by each LED, respectively. Figure 3c shows the final light intensity emitted by each LED that must be within the corresponding LED’s linear dynamic range. Furthermore, the average optical value of each color LED is selected to guarantee a specific color constraint, as we will see in Section 3.

The discrete-time received signal can be expressed as

(3) y=Hx+n,

where $H={\left[h_{1},h_{2},\ldots ,h_{D}\right]}^{T}\in R^{D\times L}$ can be further expressed as

(4) $$H=G⊗\tilde{H}$$

with $\tilde{H}\in R^{K\times N}$ being a matrix that contains the downlink channel from the *N* LEDs to the *K* users and $G\in R^{3\times 3}$ representing the cross-responsivities matrix:

(5) $$G=\left(\begin{array}{ccc} g_{R,R} & g_{R,G} & g_{R,B}\\ g_{G,R} & g_{G,G} & g_{G,B}\\ g_{B,R} & g_{B,G} & g_{B,B} \end{array}\right)$$

The element $g_{i,j}$ in G represents the optical front-end gain between the transmit band *i* and the receive band *j* that characterizes the optical-to-electrical (O/E) conversion. It is defined as

(6) $$g_{i,j}=\int_{0}^{\infty }S_{t}^{i}\left(\lambda \right)\Gamma_{r}^{j}\left(\lambda \right)d\lambda \;\;\left[A/W\right]$$

with $S_{t}^{i}\left(\lambda \right)$ the emission spectrum per Watt of the LED of the *i*-th color and $\Gamma_{r}^{j}\left(\lambda \right)$ the sensitivity of the PD to the *j*-th color [16,21].

The elements of matrix $\tilde{H}$ are non-negative and correspond to the channel gain between the *n*-th transmitter and the *k*-th receiver [31]:

(7) $${\tilde{h}}_{n,k}=\left\{\begin{array}{cc} \frac{\left(m+1\right)A_{r}}{2\pi D_{n,k}^{2}}\cos^{m}\left(\beta \right)\cos\left(\alpha \right)T_{s}\left(\alpha \right)g_{o}\left(\alpha \right) & 0\leq \alpha \leq FOV\\ 0 & \alpha >FOV. \end{array}\right.$$

As described in [9,31], the variables that appear in (7) correspond to the following parameters; the order of the Lambertian source (*m*), the effective area of the receiver ($A_{r}$), the distance between the *n*-th RGB LED and the *k*-th RGB PD ($D_{m,k}$), the irradiation angle (β), the incident angle (α), the gain of the optical filter ($T_{s}\left(\alpha \right)$), the gain of the optical concentrator ($g_{o}\left(\alpha \right)$), and the field of view (FOV) of the PDs. The order of the Lambertian emission can be further expressed as

(8) $$m=-\frac{\ln(2)}{\ln(\cos\left(\Phi_{1/2}\right))},$$

with $\Phi_{1/2}$ being the transmitter semi-angle at half-power. For a Lambertian source, $g_{o}\left(\alpha \right)$ is given by

(9) $$g_{o}\left(\alpha \right)=\left\{\begin{array}{cc} \frac{\kappa^{2}}{\sin^{2}\left(FOV\right)} & 0\leq \alpha \leq FOV\\ 0 & \alpha >FOV, \end{array}\right.$$

where κ is the refractive index of the concentrator.

The receiver noise contains thermal and shot noise. We consider that the thermal noise is white and Gaussian distributed with zero mean and variance equal to

(10) $$\sigma_{thermal}^{2}=\frac{8\pi k_{B}T_{k}}{G_{ol}}C_{pd}A_{r}I_{2}B^{2}+\frac{16\pi^{2}k_{B}T_{k}\eta }{g_{m}}C_{pd}^{2}A_{r}^{2}I_{3}B^{3}.$$

Following the same notation as the work in [9], in (10) $k_{B}$ and $T_{k}$ represent to the Boltzmann’s constant and the absolute temperature, respectively. The rest of the parameters are the open-loop voltage gain ($G_{ol}$), the capacitance per unit area of the PD ($C_{pd}$), the noise bandwidth factors ($I_{2}$ and $I_{3}$), the equivalent noise bandwidth of the PD (*B*), and the transconductance and channel noise of the field effect transistor ($g_{m}$ and η).

The variance of the shot noise is [9,31]

(11) $$\sigma_{shot}^{2}=2qI_{r}B+2qI_{B}I_{2}B$$

with *q* is the electron charge and $I_{r}$ the received current after the O/E conversion. We can compute $I_{r}$ by multiplying the received optical power by the receiving PD’s responsivity. Finally, the photocurrent coming from background radiation is denoted as $I_{B}$. From (10) and (11), we observe that the thermal noise is independent of the received optical power, but the shot noise is not. When the background radiation dominates the shot noise, for instance, in the presence of strong sunlight, the term depending on $I_{r}$ in (11) is small compared to the other term, and it is safe to assume that $\sigma_{shot}^{2}\approx 2qI_{B}I_{2}B$ [9,31]. On the other hand, in the absence of significant background radiation, the shot noise may show a strong dependency on the received power, significantly challenging the precoding design. In the following, to simplify the design, we will assume the noise variance is independent of the received signal, a valid assumption if the dominant term in (11) is the second one.

Based on the above, we model the total noise as real-valued additive white Gaussian noise with zero mean and variance [32]:

(12) $$\sigma^{2}=\sigma_{thermal}^{2}+\sigma_{shot}^{2}.$$

We can further express (3) as

(13) y=HWs+Hb+n.

As we assume that channel state information (CSI) is available at the transmitter and receiver, the direct current (DC) component, i.e., Hb, can be estimated and removed at the receiver. The electrical signal-to-noise ratio (SNR) of the *d*-th stream is

(14) $$\mathrm{SNR}=\frac{|h_{d}^{T}w_{d}{|}^{2}P_{s,\;elec}}{\sigma_{d}^{2}}.$$

We are interested in finding an expression of the achievable rate, *r*, measured in bps/Hz. In [33], the authors provide an upper yet extremely tight bound of the BER for a *M*-PAM modulation, valid for BER <0.1:

(15) $$\mathrm{BER}\leq 0.1\exp\left[-\frac{1.5\;\mathrm{SNR}}{M^{2}-1}\right].$$

Plugging (14) in this approximation we obtain the following upper bound,

(16) $$\mathrm{BER}\leq 0.1\exp\left[-\frac{1.5\;|h_{d}^{T}w_{d}{|}^{2}P_{s,\;elec}}{\sigma_{d}^{2}\left(M^{2}-1\right)}\right].$$

From (16), using the value of $P_{s,elec}$ in (2) and considering $M=2^{r_{d}},$ we obtain the following achievable rate bound,

(17) $$r_{d}\geq \log_{2}\left(1+o_{d}\left|h_{d}^{T}w_{d}\right|\right)$$

where $o_{d}=\frac{1}{\sigma_{d}\;\sqrt{-2\ln\left(10\;{\mathrm{BER}}_{\mathrm{MPAM}}\right)}}$, with ${\mathrm{BER}}_{\mathrm{MPAM}}$ being the target BER. This target BER is considered less than 0.1, so previous expressions are valid.

## 3. Optical and Electrical Constraints

Each RGB-LED must satisfy three constraints in the optical transmission. The first constraint ensures the white color of the emitted light on average. The second constraint ensures that the signal emitted by each LED is positive and within the linear dynamic range of the LED to limit nonlinear distortion. Moreover, it limits the maximum instantaneous optical power emitted. Finally, the last constraint ensures the eye safety by restricting the maximum average radiated power of our VLC system. In the following, we detail how to formulate these constraints.

### 3.1. White Color Constraint

Each RGB unit must transmit white light on average over time or, equivalently, over symbols. The first-order moment of the transmitted signal x is

(18) $$E\left\{x\right\}=b={\left[b_{R}^{T},b_{G}^{T},b_{B}^{T}\right]}^{T}.$$

Then, the DC bias applied to each LED within the *n*-th set depends on the set of primaries R, G, B chosen to guarantee white light. As a result,

(19) $$\mathrm{White}\;\mathrm{light}\;\mathrm{constraint}:\;b=\left(\begin{array}{c} b_{R}\\ b_{G}\\ b_{B} \end{array}\right)=\beta \left(\begin{array}{c} \rho_{R}\\ \rho_{G}\\ \rho_{B} \end{array}\right)⊗1_{N},$$

with

(20) $$\rho_{R}+\rho_{G}+\rho_{B}=1,$$

where $\rho_{c}$ represents the percentage of the *c*-th color contribution to the white color in each RGB unit, and β is the average optical power of each RGB unit. The color percentage depends entirely on the chosen R, G, B LEDs, while β is a variable that will be optimized. Equation (20) also ensures the mitigation of the flicker by maintaining a constant average irradiated optical power [16,34].

### 3.2. Instantaneous Optical Power Constraint

The transmitted optical samples, x, must be within the linear dynamic range of the transmitter front-end [1]:

(21) $${\tilde{p}}_{\min}\leq x\leq {\tilde{p}}_{\max}$$

with ${\tilde{p}}_{\min}={\left[P_{\min,R},P_{\min,G},P_{\min,B}\right]}^{T}⊗1_{N}$ and ${\tilde{p}}_{\max}={\begin{array}{c} \left[P_{\max,R},P_{\max,G},P_{\max,B}\right] \end{array}}^{T}⊗1_{N}$, where $P_{\min,c}$ and $P_{\max,c}$ are the minimum and maximum instantaneous optical power for the LEDs of color *c*, respectively. Note that ${\tilde{p}}_{\min},{\tilde{p}}_{\max}\in R^{3N\times 1}$. The lower bound of (21) is a generalization of the non-negativity constraint of the VLC systems. The waveform transmitted by each LED, i.e., $x_{l}$, represents optical power and, thus, needs to be real and non-negative:

(22) $$x_{l}=\sum_{d=1}^{D}w_{l,d}s_{d}+b_{l}\geq {\tilde{p}}_{\min_{l}},\;l=1,\ldots ,L.$$

when the most restrictive case is taken, i.e., when the left side of the inequality (22) is minimal ($w_{l,d}s_{d}=-\left|w_{l,d}\right|\;\forall l,d$), the following constraint can be considered.

(23) $$\sum_{d=1}^{D}\left|w_{l,d}\right|\leq b_{l}-{\tilde{p}}_{\min_{l}},\;l=1,\ldots ,L.$$

On the other hand, the upper bound restricts the individual per-LED instantaneous transmitted optical power for eye-safety reasons [35]. The peak optical power needs to be limited:

(24) $$\sum_{d=1}^{D}w_{l,d}s_{d}+b_{l}\leq {\tilde{p}}_{\max_{l}},\;l=1,\ldots ,L,$$

that, similarly, leads to

(25) $$\sum_{d=1}^{D}\left|w_{l,d}\right|\leq {\tilde{p}}_{\max_{l}}-b_{l},\;l=1,\ldots ,L.$$

(23) and (25) can be combined in three different constraints that depend mainly on the color of the LEDs:

(26) $$\begin{array}{c} \sum_{d=1}^{D}\left|w_{l,d}\right|\leq f_{R}\left(\beta \right)\;\mathrm{for}\;l=1,...,N\\ \sum_{d=1}^{D}\left|w_{l,d}\right|\leq f_{G}\left(\beta \right)\;\mathrm{for}\;l=N+1,...,2N\\ \sum_{d=1}^{D}\left|w_{l,d}\right|\leq f_{B}\left(\beta \right)\;\mathrm{for}\;l=2N+1,...,3N \end{array}$$

where $f_{c}\left(\beta \right)=\min\left(P_{\max,c}-\beta \rho_{c},\beta \rho_{c}-P_{\min,c}\right)$ and c={R,G,B} or, equivalently, in a single constraint:

(27) $$\begin{array}{c} \mathrm{Instantaneous}\;\mathrm{optical}\\ \mathrm{power}\;\mathrm{constraint}: \end{array}\sum_{d=1}^{D}\left|e_{l}^{T}w_{d}\right|\leq \min\left\{e_{l}^{T}\left({\tilde{p}}_{\max}-b\right),e_{l}^{T}\left(b-{\tilde{p}}_{\min}\right)\right\}\;\mathrm{for}\;l=1,...,L.$$

In previous expression, $e_{l}$, is a vector with all the elements equal to zero except the one in the *l*-th position that is equal to 1, i.e., $e_{l}={\left(0,...,0,1,0,...,0\right)}^{T}$.

### 3.3. Average Radiated Optical Power Constraint

Eye safety regulations constrain the level of average radiated optical power by each RGB-LED [36,37]. Consequently, the average radiated power must satisfy

(28) $$b_{R}+b_{G}+b_{B}\leq {\tilde{P}}_{\mathrm{ave}}1_{N}$$

which implies

(29) $$\mathrm{Average}\;\mathrm{optical}\;\mathrm{power}\;\mathrm{constraint}:\;\beta \leq {\tilde{P}}_{\mathrm{ave}}$$

where ${\tilde{P}}_{\mathrm{ave}}$ is the maximum average power allowed.

## 4. RGB-LED Based Zero Forcing Precoding

In this section, we focus on the design of ZF precoders for an RGB VLC system. We will start by designing a zero-forcing approach based on the pseudoinverse while considering all the previously described constraints. Our goal, however, is to develop a ZF precoder that cancels interference and maximizes the sum rate. We will undertake such a design by solving an optimization problem with the weighted sum rate as the optimization criteria. Therefore, we will refer to the resulting approach as the optimum precoder, understanding such optimality in the sum rate. In the simulations section, we will compare both schemes.

### 4.1. Zero Forcing Precoding with Pseudoinverse

The simplest ZF precoder design is the pseudoinverse of the channel, i.e., $W=\alpha H^{†}$ being α a scale factor and $H^{†}=\left[t_{1},t_{2},\ldots ,t_{D}\right]\in R^{L\times D}$ the pseudoinverse of H. Taking into account the structure of the channel matrix (4) and applying the properties of the Kronecker product, we can express the precoding matrix as

(30) $$W=\alpha H^{†}=\alpha \left(G^{†}⊗{\tilde{H}}^{†}\right).$$

Combining (17) and (30), the rate bound associated to the *d*-th PD is given by

(31) $$r_{d}\geq \log_{2}\left(1+o_{d}\left|\alpha h_{d}^{T}t_{d}\right|\right).$$

We want to maximize (31) by adjusting the value of the scale factor α and ensuring the fulfillment of the constraints described in Section 3. By enforcing the bias structure specified in (19), we ensure the white color of the emitted light. We need to select α and β that maximize (31), which is equivalent to maximize α while satisfying (26) and (29). Then, we can formulate the problem as

$$\begin{array}{cccc} \; & (\mathrm{PA}1): & \underset{\alpha ,\beta \leq {\tilde{P}}_{\mathrm{ave}}}{\;\mathrm{maximize}\;} & \alpha \\ \; & \; & s.t.\; & \sum_{d=1}^{D}\left|w_{l,d}\right|\leq f_{R}\left(\beta \right),\;\mathrm{for}\;l=1,...,N\\ \; & \; & & \sum_{d=1}^{D}\left|w_{l,d}\right|\leq f_{G}\left(\beta \right),\;\mathrm{for}\;l=N+1,...,2N\\ \; & \; & & \sum_{d=1}^{D}\left|w_{l,d}\right|\leq f_{B}\left(\beta \right),\;\mathrm{for}\;l=2N+1,...,3N \end{array}$$

Note that $\sum_{d=1}^{D}|w_{n+(c-1)N,d}\left|=\alpha (|\right|f_{c}^{T}G^{†}{\left|\right|}_{1}\cdot ||g_{n}^{T}H^{†}{\left|\right|}_{1})$ where $f_{c}$ is a 3×1 vector with all zeros but a 1 in the *c*-th position and $g_{n}$ is a N×1 vector with all zeros but a 1 in the *n*-th position. Thus, we can rewrite (PA1) as

$$\begin{array}{cccc} \; & (\mathrm{PA}2): & \underset{\alpha ,\beta \leq {\tilde{P}}_{\mathrm{ave}}}{\;\mathrm{maximize}\;} & \alpha \\ \; & \; & s.t.\; & \left|\right|g_{n}^{T}H^{†}{\left|\right|}_{1}\alpha \leq \frac{f_{R}\left(\beta \right)}{\left|\right|f_{1}^{T}G^{+}{\left|\right|}_{1}},\;\mathrm{for}\;n=1,...,N\\ \; & \; & & \left|\right|g_{n}^{T}H^{†}{\left|\right|}_{1}\alpha \leq \frac{f_{G}\left(\beta \right)}{\left|\right|f_{2}^{T}G^{+}{\left|\right|}_{1}},\;\mathrm{for}\;n=1,...,N\\ \; & \; & & \left|\right|g_{n}^{T}H^{†}{\left|\right|}_{1}\alpha \leq \frac{f_{B}\left(\beta \right)}{\left|\right|f_{3}^{T}G^{+}{\left|\right|}_{1}},\;\mathrm{for}\;n=1,...,N \end{array}$$

The value of β that achieves the greatest α is the one that maximizes the worst one of the three bounds (because the term $\left|\right|g_{n}^{T}H^{†}{\left|\right|}_{1}$ is common in the three groups of inequalities):

(32) $$\beta^{*}=\underset{\beta \leq \tilde{P}\mathrm{ave}}{\mathrm{argmax}\;}\left(\min\left\{\frac{f_{R}\left(\beta \right)}{\left|\right|f_{1}^{T}G^{+}{\left|\right|}_{1}},\frac{f_{G}\left(\beta \right)}{\left|\right|f_{2}^{T}G^{+}{\left|\right|}_{1}},\frac{f_{B}\left(\beta \right)}{\left|\right|f_{3}^{T}G^{+}{\left|\right|}_{1}}\right\}\right)$$

We observe that $\beta^{*}$ does not depend on either the number of users or the particular channels. It only depends on matrix G, $P_{\max,R/G/B}$, $P_{\min,R/G/B}$, and $\rho_{R/G/B}$, and therefore it can be computed beforehand. Therefore, the optimum bias vector is

(33) $$b^{*}=\left(\begin{array}{c} \rho_{R}\\ \rho_{G}\\ \rho_{B} \end{array}\right)⊗1_{N}\beta^{*},$$

and the optimum scaling factor, $\alpha^{*}$, is

(34) $$\alpha^{*}=\frac{\min\left\{\frac{f_{R}\left(\beta^{*}\right)}{\left|\right|f_{1}^{T}G^{+}{\left|\right|}_{1}},\frac{f_{G}\left(\beta^{*}\right)}{\left|\right|f_{2}^{T}G^{+}{\left|\right|}_{1}},\frac{f_{B}\left(\beta^{*}\right)}{\left|\right|f_{3}^{T}G^{+}{\left|\right|}_{1}}\right\}}{\max_{n}\left\{|\right|g_{n}^{T}H^{+}{\left|\right|}_{1}\}}.$$

### 4.2. Optimal Zero Forcing Precoding for Maximum Weighted Sum Rate

As a starting point, we are going to reconsider the rate bound in (17):

(35) $$r_{d}\geq \log_{2}\left(1+o_{d}\left|h_{d}^{T}w_{d}\right|\right)$$

The precoding matrix needs to satisfy the optical power constraints defined in Section 3. We consider $b=b^{*}$ (33) ensuring the fulfillment of (19) and (29). In addition, the ZF constraint $h_{j}^{T}w_{d}=0,\;\forall j\neq d$ is imposed.

We can formulate the problem as follows,

$$\begin{array}{cccc} \; & (\mathrm{PB}1): & \underset{\left\{w_{d}\right\}}{\;\mathrm{maximize}\;} & \sum_{d=1}^{D}u_{d}\log_{2}\left(1+o_{d}\left|h_{d}^{T}w_{d}\right|\right)\\ \; & \; & s.t.\; & h_{j}^{T}w_{d}=0,\;\forall j\neq d\\ \; & \; & & \sum_{d=1}^{D}\left|e_{l}^{T}w_{d}\right|\leq \min\left(e_{l}^{T}\left({\tilde{p}}_{\max}-b\right),e_{l}^{T}\left(b-{\tilde{p}}_{\min}\right)\right),\;\mathrm{for}\;l=1,...,L, \end{array}$$

where $u_{d}$ is a weighting factor for the rate of the *d*-th stream.

Note that the precoder design depends on the noise variance, which needs to be estimated along with the channel. As the precoder affects the received power, the difficulty comes if the noise variance depends on the received power. Nevertheless, under the assumption that the shot noise’s dominant term is due to the background radiation, the noise variance is practically independent of the received signal.

The composite function $\log_{2}\left(1+o_{d}\left|h_{d}^{T}w_{d}\right|\right)$ is not concave w.r.t. $w_{d}$. As (PB1) aims to maximize this function, (PB1) is not either a concave or convex problem. However, we may rewrite (PB1) as an equivalent concave problem that achieves the same solution. To that end, we add the constraint $h_{d}^{T}w_{d}\geq 0\;\forall d$ and remove the absolute value in the objective function. As explained in [9], where we used the same method to solve a problem with the same structure (problem PA1 in [9]), adding the new constraint does not imply a loss of optimality. Indeed, if there is an optimum $w_{d}$ such as $h_{d}^{T}w_{d}\leq 0$, then $-w_{d}$ achieves the same value of the objective function and fulfill the set of constraints, including the new one. Summarizing, (PB1) is equivalent to the following concave problem (PB2),

$$\begin{array}{cccc} \; & (\mathrm{PB}2): & \underset{\left\{w_{d}\right\}}{\;\mathrm{maximize}\;} & \sum_{d=1}^{D}u_{d}\log_{2}\left(1+o_{d}h_{d}^{T}w_{d}\right)\\ \; & \; & s.t. & h_{j}^{T}w_{d}=0,\;\forall j\neq d\\ \; & \; & & \sum_{d=1}^{D}\left|e_{l}^{T}w_{d}\right|\leq \min\left(e_{l}^{T}\left({\tilde{p}}_{\max}-b\right),e_{l}^{T}\left(b-{\tilde{p}}_{\min}\right)\right),\;\mathrm{for}\;l=1,...,L,\\ \; & \; & & h_{d}^{T}w_{d}\geq 0,\;\mathrm{for}\;d=1,...,D. \end{array}$$

Being (PB2) a concave problem implies that we can solve it with standard optimization tools [38]. Such tools as, for example, the Lagrange duality method compute the solution numerically with affordable computational complexity. Additionally, to simplify the computation and gain some intuition on the solution of problem (PB2), we may define a matrix $X_{d}={\left[h_{1},...,h_{d-1},h_{d+1},...,h_{D}\right]}^{T}\in R^{(D-1)\times L}$, we compute its SVD, $X_{d}=U_{d}\Sigma_{d}V_{d}^{T}$ and we define the projection matrix $F_{d}=I-V_{d}V_{d}^{T}={\tilde{V}}_{d}{\tilde{V}}_{d}^{T}$. The matrix ${\tilde{V}}_{d}\in R^{L\times (L-D+1)}$ contains the vectors of the subspace orthogonal to the interference and it satisfies that $V_{d}^{T}{\tilde{V}}_{d}=0$ and ${\tilde{V}}_{d}^{T}{\tilde{V}}_{d}=I$. If we force the precoding vector, $w_{d}\in R^{L\times 1}$, to be a linear combination of the orthogonal vectors to the interference, i.e., $w_{d}={\tilde{V}}_{d}q_{d}$ where $q_{d}\in R^{(L-D+1)\times 1}$, we can remove the ZF constraint as $X_{d}w_{d}=\left(U_{d}\Sigma_{d}V_{d}^{T}\right){\tilde{V}}_{d}q_{d}=0$. Then, (PB2) can be rewritten as follows,

$$\begin{array}{cccc} \; & (\mathrm{PB}3): & \underset{\left\{q_{d}\right\}}{\;\mathrm{maximize}\;} & \sum_{d=1}^{D}u_{d}\log_{2}\left(1+o_{d}h_{d}^{T}{\tilde{V}}_{d}q_{d}\right)\\ \; & \; & s.t.\; & \sum_{d=1}^{D}\left|e_{l}^{T}{\tilde{V}}_{d}q_{d}\right|\leq \min\left(e_{l}^{T}\left({\tilde{p}}_{\max}-b\right),e_{l}^{T}\left(b-{\tilde{p}}_{\min}\right)\right),\;\mathrm{for}\;l=1,...,L\\ \; & \; & & h_{d}^{T}{\tilde{V}}_{d}q_{d}\geq 0,\;\mathrm{for}\;d=1,...,D. \end{array}$$

As (PB3) is concave, we can also find the solution of (PB3) through standard optimization tools. The solution of (PB2) and (PB3) will be, of course, the same. The computation, however, will be more straightforward for (PB3) as it has a smaller number of constraints and the dimensions of the optimization variables, i.e., $\left\{q_{d}\right\}$ are reduced compared to $\left\{w_{d}\right\}$.

## 5. PWLED Based Zero Forcing Precoding

In this section, we particularize the developed solutions to a PWLED-based system that sends only one stream per user. Instead of considering L=3N color LEDs and D=3K streams, each transmission unit has a single phosphor-coated white LED, i.e., L=N, and each user is equipped with a single PD, receiving only one stream, i.e., D=K. In this case, the transmitted signal can be expressed as follows,

(36) x=Ws+b,

where **$x\in R^{L\times 1}$, $W\in R^{L\times K}$** is the precoding matrix, $s\in R^{K\times 1}$ is the information-bearing signal, and $b\in R^{L\times 1}$ is the bias vector. The received discrete-time baseband signal is

(37) y=γHx+n,

where $y\in R^{K\times 1}$, γ is the responsivity of a white LED, $H={\left[h_{1},h_{2},\ldots ,h_{K}\right]}^{T}\in R^{K\times L}$, and $n\in R^{K\times 1}$. Note that, unlike the RGB system (4), matrix H contains only the DC channel gains and the responsivity is a scalar instead of a 3×3 matrix as only one band is used.

The instantaneous optical constraint (27) can be equivalently defined for the PWLED based system as follows,

(38) $$\mathrm{Instantaneous}\;\mathrm{optical}\;\mathrm{power}\;\mathrm{constraint}:\;\sum_{k=1}^{K}\left|w_{l,k}\right|\leq P_{l},\;l=1,\ldots ,L$$

where $P_{l}=\min\left(b_{l}-P_{\min,l},\;P_{\max,l}-b_{l}\right)$ with $P_{\min,l}$ and $P_{\max,l}$ being the maximum and minimum instantaneous optical power for the *l*-th LED, respectively.

Regarding the average optical transmitted power by the *l*-th LED, the following constraint must be satisfied.

(39) $$\mathrm{Average}\;\mathrm{optical}\;\mathrm{power}\;\mathrm{constraint}:\;E\left\{x_{l}\right\}=b\leq {\tilde{P}}_{ave}.$$

Note that it is reasonable to consider $P_{\max,l}=P_{\max}$ and $P_{\min,l}=P_{\min}$ for l=1,…,L. Then, (38) is enlarged when $b_{l}=\frac{P_{\max}-P_{\min}}{2}\;\forall l$. Considering $\frac{P_{\max}-P_{\min}}{2}\leq {\tilde{P}}_{\mathrm{ave}}$, the optimum value of $b_{l}$ is

(40) $$b_{l}^{*}=\frac{P_{\max}-P_{\min}}{2}\;\forall l.$$

Then, we can rewrite (38) and (39) in a single constraint as

(41) $$\mathrm{Optical}\;\mathrm{power}\;\mathrm{constraint}:\;\sum_{k=1}^{K}\left|e_{l}^{T}w_{k}\right|\leq \frac{P_{\max}-P_{\min}}{2}.$$

Finally, the BER bound (16) must be rewritten accordingly to the definition of the channel matrix:

(42) $$\mathrm{BER}\leq 0.1\exp\left[-\frac{1.5\gamma^{2}{\left|h_{k}^{T}w_{k}\right|}^{2}P_{s,\;elec}}{\sigma_{k}^{2}\left(M^{2}-1\right)}\right],$$

leading to the following rate bound,

(43) $$r_{k}\geq \log_{2}\left(1+o_{k}\left|h_{k}^{T}w_{k}\right|\right),$$

for k=1,…,K, where now

(44) $$o_{k}=\frac{\gamma }{\sigma_{k}\sqrt{-2\ln\left(10\;{\mathrm{BER}}_{\mathrm{MPAM}}\right)}}.$$

### 5.1. Zero Forcing Precoding with Pseudoinverse

Similarly to Section 4.1, we take the pseudoinverse of the channel matrix as precoder $W=\alpha H^{†}=\alpha \tilde{W}$ where α is the scale factor of the precoding matrix that should be selected to maximize (43) while satisfying (41), that can be rewritten as

(45) $$\sum_{k=1}^{K}\left|{\tilde{w}}_{l,k}\right|\leq \frac{P_{\max}-P_{\min}}{2\alpha },\;\mathrm{for}\;l=1,\ldots ,L.$$

Then, to maximize the rate while satisfying (45), we need to choose $\alpha^{*}=\frac{P_{\max}-P_{\min}}{2\cdot \max_{l}\left\{\sum_{k=1}^{K}\left|{\tilde{w}}_{l,k}\right|\right\}}.$

### 5.2. Optimal Zero Forcing Scheme for Maximum Weighted Sum-Rate

For PWLEDs, there is no need to consider a color constraint, and each user receives only one stream. Moreover, as we have already seen in this section, the instantaneous and average optical power constraints can be written as the single optical power constraint (41). Thus, the concave problem (PB3) can be simplified as follows,

$$\begin{array}{cccc} \; & (\mathrm{PC}3): & \underset{\left\{q_{k}\right\}}{\;\mathrm{maximize}\;} & \sum_{k=1}^{K}u_{k}\log_{2}\left(1+o_{k}h_{k}^{T}{\tilde{V}}_{k}q_{k}\right)\\ \; & \; & s.t.\; & \sum_{k=1}^{K}\left|e_{l}^{T}{\tilde{V}}_{k}q_{k}\right|\leq \frac{P_{\max}-P_{\min}}{2},\;\mathrm{for}\;l=1,...,L,\\ \; & \; & & h_{k}^{T}{\tilde{V}}_{k}q_{k}\geq 0,\;\mathrm{for}\;k=1,...,K, \end{array}$$

with $o_{k}$ including the responsitivity, as defined in Equation (44), and matrix H containing only the DC channel gains.

## 6. Numerical Results

This section presents numerical results for the precoders in previous sections, with an adaptive modulation strategy that balances the constellation size to achieve a target BER. This strategy allows high-speed transmissions under favorable channel conditions while reducing the rate when the conditions degrade. In the next experiments, the transmitted power and the modulation are adjusted to obtain an uncoded BER under $10^{-6}$ for each stream. For that purpose, using Equations (16) and (42) for RGB-LED- and PWLED-based systems, respectively, we calculate the BER for each SNR when using different constellation sizes, i.e., $2^{M}$. Then, we select the highest *M* for each user while ensuring a BER under the target one.

Table 2 contains the simulation parameters. The bandwidth of color LEDs is usually between 10 and 20 MHz [15,16]. Accordingly, we will assume a bandwidth of 15 MHz for the color LEDs, and 2 MHz for the phosphor-coated LEDs [39]. To make a fair comparison, we have also considered a bandwidth of 15MHz for the PWLEDs, which could be achieved by suppressing the slow response of the phosphorescent component of the LED but increasing the cost significantly [40]. Even if impractical, this experiment will allow us to extract fair conclusions about the performance of PWLEDs and RGB-LEDs regardless of the bandwidth.

We have considered two different sets of primaries RGB, with different primaries’ center wavelengths, for illustration purposes. Each primary can be described by two chromaticity parameters *x* and *y* in the color space chromaticity diagram CIE 1931 [41]. The chosen red, green, and blue transmitters form a triangle that encloses the colors generated by combining the three color sources. The human eye can actually perceive a range of lights without a discernible difference in color, given by MacAdam ellipses or, in the case of LED products, quadrangles, instead of ellipses, centered at a reference point [42]. However, when combining the three light sources, we will force a stable color transmission as in [17], section 12.8. Therefore, each RGB unit at the transmitter (i.e., one red, one green, and one blue LED co-located together) always generates light with specific color coordinates. Note that, if a broader region of color coordinates for the target white light were allowed, then the problem would be less constrained, and the achievable rates could potentially be improved.

**Figure 4:**
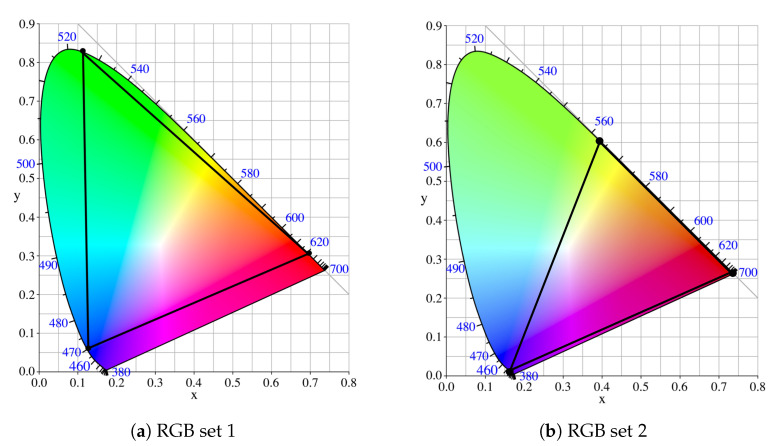
RGB primaries sets considered in simulations.

The selected primaries (i.e., R, G, B lights) have been taken from the works in [16,17], although any set of R, G, B primaries is possible for the VLC system design. The two chosen RGB sets and the respective color proportions are specified in Table 3 and represented in Figure 4. From now on, the first or the second sets will be referred to as RGB-LED 1 and RGB-LED 2, respectively.

Given the spectrum of a light, Λ(λ), the XYZ coordinates can be computed from the CIE 1931 tri-stimulus functions as follows [43],

(46) $$\begin{array}{c} X=K_{m}\int_{0}^{\infty }\Lambda \left(\lambda \right)\bar{x}\left(\lambda \right)d\lambda ,\\ Y=K_{m}\int_{0}^{\infty }\Lambda \left(\lambda \right)\bar{y}\left(\lambda \right)d\lambda ,\\ Z=K_{m}\int_{0}^{\infty }\Lambda \left(\lambda \right)\bar{z}\left(\lambda \right)d\lambda , \end{array}$$

where $K_{m}$, the maximum luminous efficacy, is 683 lm·$W^{-1}$. From Equation (46) and the tri-stimulus data in [43], we can compute the XYZ coordinates for each one of the primaries considering, for example, unitary power. Then, adjusting the amounts of energy of the monochromatic lights according to $\rho_{R}$, $\rho_{G}$, and $\rho_{B}$, and adding the three lights, we have

(47) $$\begin{array}{c} X_{c}=\rho_{R}X_{R}+\rho_{G}X_{G}+\rho_{B}X_{B},\\ Y_{c}=\rho_{R}Y_{R}+\rho_{G}Y_{G}+\rho_{B}Y_{B},\\ Z_{c}=\rho_{R}Z_{R}+\rho_{G}Z_{G}+\rho_{B}Z_{B}, \end{array}$$

and the chromaticities coordinates of the combined light are

(48) $$\left(x_{c},y_{c},z_{c}\right)=\left(\frac{X_{c}}{X_{c}+Y_{c}+Z_{c}},\frac{Y_{c}}{X_{c}+Y_{c}+Z_{c}},\frac{Z_{c}}{X_{c}+Y_{c}+Z_{c}}\right)$$

For the values in Table 3, this computation results in $\left(x_{c},y_{c}\right)\approx \left(0.3,0.33\right)$ for both sets. The color, therefore, is very close to the CIE Standard Illuminant D65 [44] whose chromaticity parameters are $\left(x_{W},y_{W}\right)=\left(0.31271,0.32902\right)$. Note that any further equal scaling, β, of the power of the three primary lights will not change $\left(x_{c},y_{c}\right)$.

**Figure 5:**
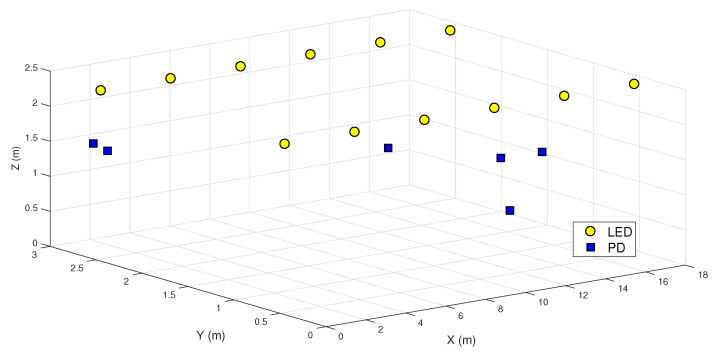
Distribution of LEDs and PDs in a 3D space.

The deployment of LEDs and users considered for the simulations is motivated by the rail wagon scenario described in Section 1. While the LEDs’ positions in all the simulations correspond to the ones shown in Figure 5, the number of users and their positions may change. As explained in Section 2, when designing the precoders, we assume that the second term in Equation (11) dominates the shot noise variance, which is, therefore, practically independent of the received power. In the scenario considered, a train carriage, this assumption is plausible if sunlight is entering the wagon. Nevertheless, after designing the precoders, we evaluate their performance considering all the noise terms, including the term in the shot noise that depends on the received power.

Note that, at the transmitter side, the transmission power of the three primary sources ensures specific CIE 1931 xy coordinates that account for the human color perception. At the receiver side, we compute the received intensity from the received power and the responsivity of the photodetectors (not the human eye). The cross-responsivity matrix depicted in Table 2 accounts for the optical-electrical conversion efficiency depending on the LED’s wavelength and the sensitivity of the PDs used. To use reasonable/realistic values in the simulation sections, we have considered the responsivity values presented in [16] for a specific combination of LEDs and PDs.

**Figure 6:**
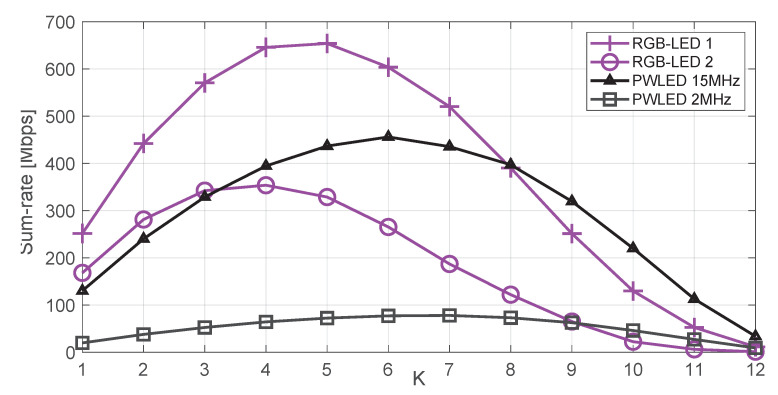
Sum rate versus the number of users for the pseudo-inverse precoder. “RGB-LED” refers to a system with *N* = 12 RGB units, each unit composed of one red, one green, and one blue LED colocated together. “PWLED” refers to a system with twelve phosphor-coated LEDs.

Figure 6 shows the average sum-rate for different values of *K* (number of users) considering the pseudoinverse precoder (derived in Section 4.1 for an RGB-based system and in Section 5.1 for a PWLED-based system). The results correspond to $10^{4}$ random realizations. For each independent run, the LED’s distribution is shown in Figure 5, while the users’ positions are random. Figure 6 shows that the sum rate does not steadily increase with the number of users. There are three reasons for this behavior: The first one is the fact that the optical power is limited. The second one is that forcing spatial zeros becomes more difficult as the number of users in a limited area increases. Finally, the inequalities in (23) and (25) account for a worst-case in which the user’s symbols add constructively in the per-LED constraint, and this assumption becomes more pessimistic and challenging as the number of users increases. Therefore, increasing the number of users will constrain the value $|w_{l,d}|$ for each specific user. As expected, the RGB-LEDs of 15 MHz outperforms the PWLEDs of 2 MHz. Figure 6 also shows the performance of a PWLED-based system of 15 MHz, so conclusions can be extracted about the performance of the two systems regardless of bandwidth. Interesting enough, the RGB-LED based system still offers a better performance up to 8–9 users. Then, when the number of users approaches the number of LEDs, the PWLED system outperforms the RGB system, as the latter has to deal with an increased level of interference and the need to force more spatial zeros.

**Figure 7:**
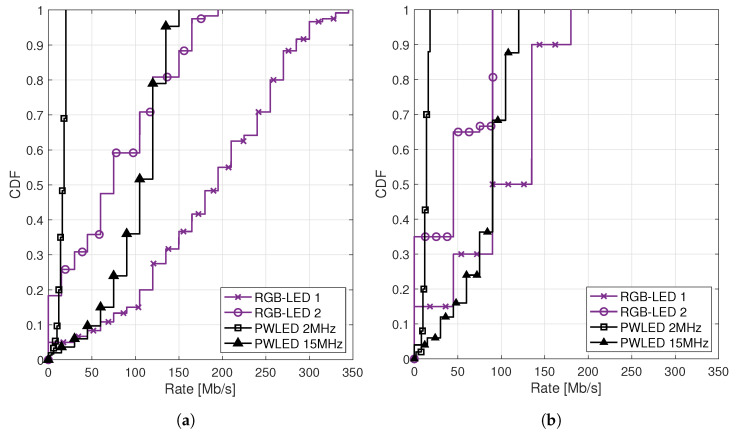
CDF of the users’ rates obtained in a system with K=6 users for the (**a**) optimal precoder and (**b**) pseudoinverse precoder. The CDFs have been obtained with 1000 independent realizations varying randomly the users’ positions. For each realization, the BER requirement is satisfied through the variation of the M-PAM constellation. “RGB-LED” refers to a system with N=12 RGB units, each unit composed of one red, one green, and one blue LED placed together. “PWLED” refers to a system with 12 phosphor-coated LEDs.

Figure 7 compares the performance of the pseudoinverse precoder (see Section 4.1 and Section 5.1) and the precoder that maximizes the weighted sum-rate, i.e., optimal precoder (see Section 4.2 and Section 5.2). To facilitate the comparison, the weights are set to 1. Figure 7 that shows the Cumulative Distribution Function (CDF) of the rates illustrates again that even if we increase the PWLED BW from 2MHz to the RGB-LEDs’ BW, i.e., 15MHz, the PWLED system presents poorer results in terms of rate. This behavior is observed for both the pseudoinverse precoder and the optimum precoder. On the other hand, the optimal precoder outperforms the pseudoinverse significantly. For instance, when considering the RGB-LEDs 1 as transmitters, the worst 50% users achieve nearly 195Mbps with the optimal precoder, while when taking the pseudoinverse they only achieve 90Mbps.

**Figure 8:**
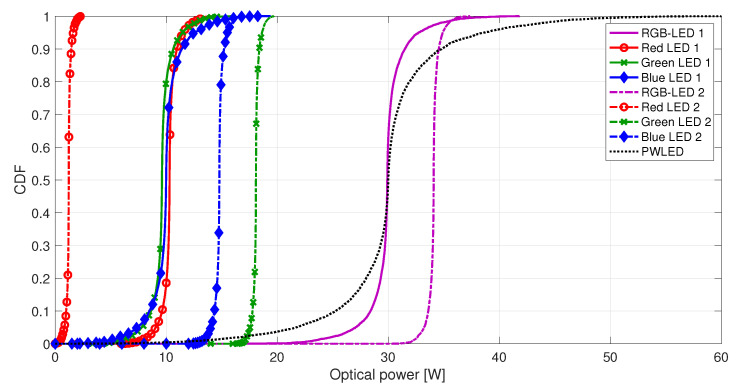
CDF of the instantaneous optical powers of RGB units, red LEDs, green LEDs, and blue LEDs in a system with N=12 RGB units, and CDF of the instantaneous optical power of PWLED LEDs in a system with twelve phosphor-coated LEDs. The number of users in the system is K=6. The CDFs have been obtained with 1000 independent realizations varying randomly the users’ positions. For each realization, the BER requirement is satisfied through the variation of the M-PAM constellation.

Another aspect observed in both Figure 6 and Figure 7 is the superior performance of the first set of primaries with respect to the second one for the RGB-LED-based system. The fact that the red channel is underused in the second set strongly penalizes the second RGB system, as can be observed in Figure 4b. Instead, the first RGB system provides similar power to the three color channels since $\rho_{R}≃\rho_{G}≃\rho_{B}$, as shown by the CDF of the instantaneous optical power per LED in Figure 8.

Finally, Table 4 presents the sum rate achieved by the proposed schemes for the particular distribution of LEDs and PDs depicted in Figure 5. The same already mentioned observations apply for this particular scenario.

## 7. Conclusions

In this paper, we have focused on the design of ZF multi-user precoding techniques for VLC systems. For the study, we have considered two types of transmitters: RGB-LEDs, which offer higher modulation bandwidth and modulate separate data streams on the three colors, and PWLEDs, which are the traditional white phosphor LEDs. For the RGB VLC system, we have carried out the precoding design by solving an optimization problem subject to a white color constraint, and instantaneous and average optical power constraints. We have developed a precoder under this approach that maximizes the sum-rate and stands out significantly from the pseudoinverse precoder.

Establishing the same average radiated optical power for the two kinds of transmitters, our results show that when adopting RGB-LEDs, the achieved user’s rates are much higher than when using PWLEDs. Even if we increase the PWLED’s BW to the RGB-LEDs’ BW by adding a blue filter in the receiver or other similar approaches, the RGB-based systems still achieve better results. However, the results depend critically on the set of selected primaries R, G, B. Although the procedure followed to design the precoders is independent of these primaries, the system performance improves when the allocated power for each of the three RGB components is similar.

## Figures and Tables

**Table 1:** Differences between VLC and RF systems [2].

	RF	VLC	Impact on VLC Systems
Bandwidth	From 3 Hz to 3000 GHz.	From 380 to 780 THz	Massive unused free bandwidth is available.
Spectrum regulation	Strict	None	Worldwide compatibility.
EM interference	Possible interference with electronic devices.	No interference with electronic devices.	VLC can be used safely in hospitals, planes, etc.
Surface penetration	It penetrates walls and other surfaces. It is strongly attenuated in water.	Can not propagate through opaque barriers but propagates through water.	Less coverage. Securely deployed. Suitable for underwater communications.
Channel model	The data-carrying signal modulates the complex- valued bipolar electric field radiated by an antenna.	The signal modulates the intensity of the optical emitter (IM/DD).	The channel input needs to be real-valued and positive. Positive channel model.
The channel input, x(t), represents	Amplitude	Optical power	
Average power proportional to	${\int |x\left(t\right)|}^{2}dt$	∫x(t)dt	

**Table 2:** Simulation parameters.

		RGB		White
**LED parameters**				
Number of transmitters	*N*	12		
Number of LEDs	*L*	36		12
Maximum transmitted optical power	$P_{max}$	*R*	20W	60W
		*G*	20W	
		*B*	20W	
Minimum transmitted optical power	$P_{min}$	*R*	0W	0W
		*G*	0W	
		*B*	0W	
Maximum average optical power	$\tilde{P}ave$	40W		
Bandwidth	BW	*R*	15MHz	2MHz
		*G*	15MHz	
		*B*	15MHz	
Semi-angle at half power	$\Phi_{1/2}$	70°		
Center luminous intensity	$I_{0}$	0.73cd		
**PD parameters**				
Number of users	*K*	6		
Field of View	FOV	60°		
Detector physical area of a PD	$A_{r}$	$1\;{\mathrm{cm}}^{2}$		
Gain of the optical filter	$T_{s}\left(\alpha \right)$	1		
Refractive index of the concentrator	κ	1.5		
Responsivity	γ	$G=\left(\begin{array}{ccc} 0.381 & 0.002 & 0\\ 0 & 0.276 & 0.024\\ 0 & 0.034 & 0.194 \end{array}\right)$ [16]		0.53A/W
**Noise parameters** noise-related parameters can be found in [31]				

**Table 3:** RGB primaries sets.

	Set 1			Set 2		
	**Wavelength**	(x,y)	ρ	**Wavelength**	(x,y)	ρ
**Red**	625nm	(0.701,0.299)	0.3444	753nm	(0.734,0.265)	0.0358
**Green**	525nm	(0.114,0.826)	0.3214	564nm	(0.402,0.594)	0.5305
**Blue**	470nm	(0.124,0.058)	0.3342	429nm	(0.169,0.007)	0.4338

**Table 4:** Sum rates achieved in [Mb/s] for K=6 users placed at the specific positions depicted in Figure 5. “RGB” refers to a system with N=12 RGB units, each unit composed of one red, one green, and one blue LED co-located together. “White” refers to a system with twelve phosphor-coated LEDs.

Precoders	RGB		White	
	Set 1	Set 2	BW=2MHz	BW=15MHz
**Optimal precoder**	1185	525	98	630
**Pseudoinverse**	810	270	84	540
